# Characterization of the complete plastome of *Dysphania botrys*, a candidate plant for cancer treatment

**DOI:** 10.1080/23802359.2018.1530964

**Published:** 2018-10-31

**Authors:** Yao Chen, Zhaoping Yang

**Affiliations:** a College of Life Sciences, Tarim University, Alaer, China;; b Laboratory of Systematic & Evolutionary Botany and Biodiversity, College of Life Sciences, Zhejiang University, Hangzhou, China

**Keywords:** *Dyphania botrys*, chloroplast genome, phylogenomics, Amaranthaceae

## Abstract

*Dysphania botrys* belongs to Amaranthaceae and distributes in North Europe, Asia, and North America. It is a medicinal plant with diuretic, antispasmodic, carminative, antidiarrhoeic properties, and a candidate plant for cancer treatment. However, few studies focused on its phylogeny, and its taxonomic status is still controversial. To better understand the evolution of this species, the complete plastome of *D. botrys* was obtained by next-generation sequencing. It is the first plastome to be sequenced and reported in the genus *Dysphania*. The plastome is 152,055 bp in length, which consists of a large single-copy region (LSC, 83,769 bp; GC content: 34.7%), a small single-copy region (SSC, 17,916 bp; GC content: 30.1%), and a pair of inverted repeat regions (IRs, 25,185 bp; GC content: 42.7%). It harbors 112 unique genes, including 78 protein-coding genes, 30 transfer RNA genes, and four ribosomal RNA genes with an overall G + C content of 36.8%. The phylogeny of Amaranthaceae based on the complete plastome sequences of 13 taxa showed that *D. botrys* belong to subfamily Chenopodioideae. Chenopodioideae, together with Betoideae formed a sister clade to the three subfamilies (Salicornioideae, Suaedoideae, and Salsoloideae), and this sister clade formed an evolutionary sister clade to Amaranthoideae. Our data will largely enrich the genetic information of *Dysphania botrys* and facilitate future studies on its evolutionary status.

*Dysphania botrys* belongs to Amaranthaceae (APG IV 2016) and distributed in North Europe, Asia, and North America. It is a medicinal plant with diuretic, antispasmodic, carminative and antidiarrhoeic properties (Zoran et al. 2005), and also an interesting novel candidate for cancer treatment (Morteza-Semnani 2015). However, few studies have been focused on its phylogeny, and its taxonomic status is still controversial (Oluwatoyin and Chase 2009; Uotila 2013). To better understand its evolution, here we reported and characterized the complete plastome of *D. botrys* and presented a phylogeny of Amaranthaceae.

Leaf samples were collected from Fuhai Forestfarm, Fuhai County, Altay City, Xinjiang, China. Voucher herbarium specimen (Yang2017002) was deposited at the Herbarium of Tarim University (TZU). Total DNA was extracted from the silica-gel dried leaf tissue using DNA Plantzol Reagent (Invitrogen, Carlsbad, USA), following the manufacturer’s protocol. Then, raw reads were obtained by next-generation sequencing, conducting on the Illumina Hiseq Platform (Illumina, San Diego, CA). The complete plastome was assembled via NOVOPlasty (Dierckxsens et al. 2017) with the plastome sequence of *Chenopodium album* (GenBank accession number: NC_034950) as a reference. The annotation was performed using Geneious 11.0.5 (Biomatters Ltd., Auckland, New Zealand). Finally, clean reads were re-mapped to the draft genome and yielded the plastome sequence of *D. botrys* (GenBank accession number: MH898873).

The complete plastome of *D. botrys* is 152,055 bp in length, consists of a large single-copy region (LSC, 83,769 bp；GC content: 34.7%), a small single-copy region (SSC, 17,916 bp; GC content: 30.1%), and a pair of inverted repeat regions (IRs, 25,185 bp; GC content: 42.7%). The plastome harbors 112 unique genes, including 78 protein-coding genes, 30 transfer RNA genes, and four ribosomal RNA genes, with an overall G + C content of 36.8%. Sixteen genes are duplicated in the IR regions, including all four rRNA genes, seven tRNA genes, and five protein-coding genes.

The phylogeny of 13 Amaranthaceae species and one outgroup taxa (*Colobanthus quitensis*, Caryophyllaceae) were reconstructed based on the complete plastome sequences, using both maximum likelihood (ML) and Bayesian inference (BI) methods. We implemented these methods on CIPRES Science Gateway V.3.3 (Miller et al. 2010). RAxML-HPC v.8.2.10 (Stamatakis 2014) and XSEDE v3.2.6 (Ronquist and Huelsenbeck 2003) were used for building ML and Mrbayes trees, respectively. Consequently, ML and BI analyses generated the same tree topology (Figure 1). The phylogeny of Amaranthaceae based on the complete plastomes of 13 taxa showed that *D. botrys* belonged to Chenopodioideae. Chenopodioideae, together with Betoideae formed a sister clade to the three subfamilies (Salicornioideae, Suaedoideae, and Salsoloideae), and this sister clade formed an evolutionary sister clade to Amaranthoideae. This result was similar to the most recent phylogenetic studies of Amaranthaceae, but with much higher bootstrap support (Fuentes-Bazan et al. 2012; Hong et al. 2017). Overall, we firstly report the complete chloroplast of genera *Dysphania*, and our data will largely enrich the genetic information of *D. botrys* and facilitate future studies on its evolutionary status.

**Figure 1.**
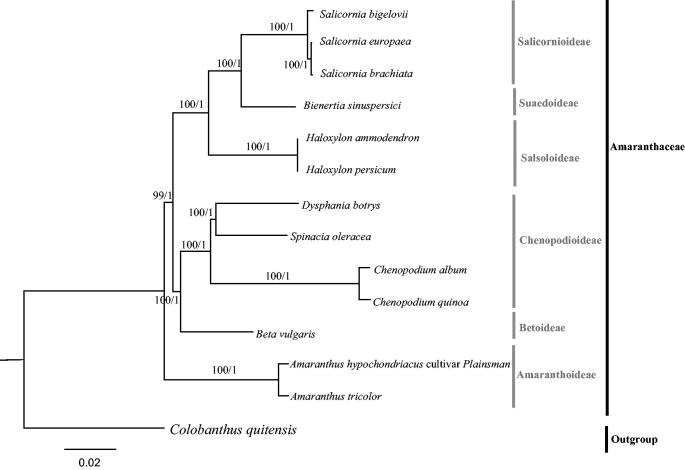
Molecular phylogeny of Amaranthaceae based on the complete plastomes of 13 taxa, with *Colobanthus quitensis* (Caryophyllaceae) as the outgroup. The accession numbers are listed as below: *Haloxylon ammodendron* (NC_027668), *Haloxylon persicum* (NC_027669), *Salicornia europaea* (NC_027225), *Salicornia brachiata* (NC_027224), *Salicornia bigelovii* (NC_027226), *Bienertia sinuspersici* (KU726550), *Beta vulgaris* var. *vulgaris* (KR230391), *Spinacia oleracea* (NC_002202), *Chenopodium album* (NC_034950), *Chenopodium quinoa* (NC_034949), *Amaranthus tricolor* (KX094399), *Amaranthus hypochondriacus* (NC030770), *Colobanthus quitensis* (KT737383).
